# Supplementary material for: Flower Buds Confirmed in the Early Cretaceous of China
Source: Biology (Basel). 2024 Jun 4;13(6):413. doi: 10.3390/biology13060413 (PMC11200749; doi:10.3390/biology13060413)
Supplement: Supplementary file 1 [file biology-13-00413-s001.zip › biology-2972331-supplementary.pdf]

Supplementary

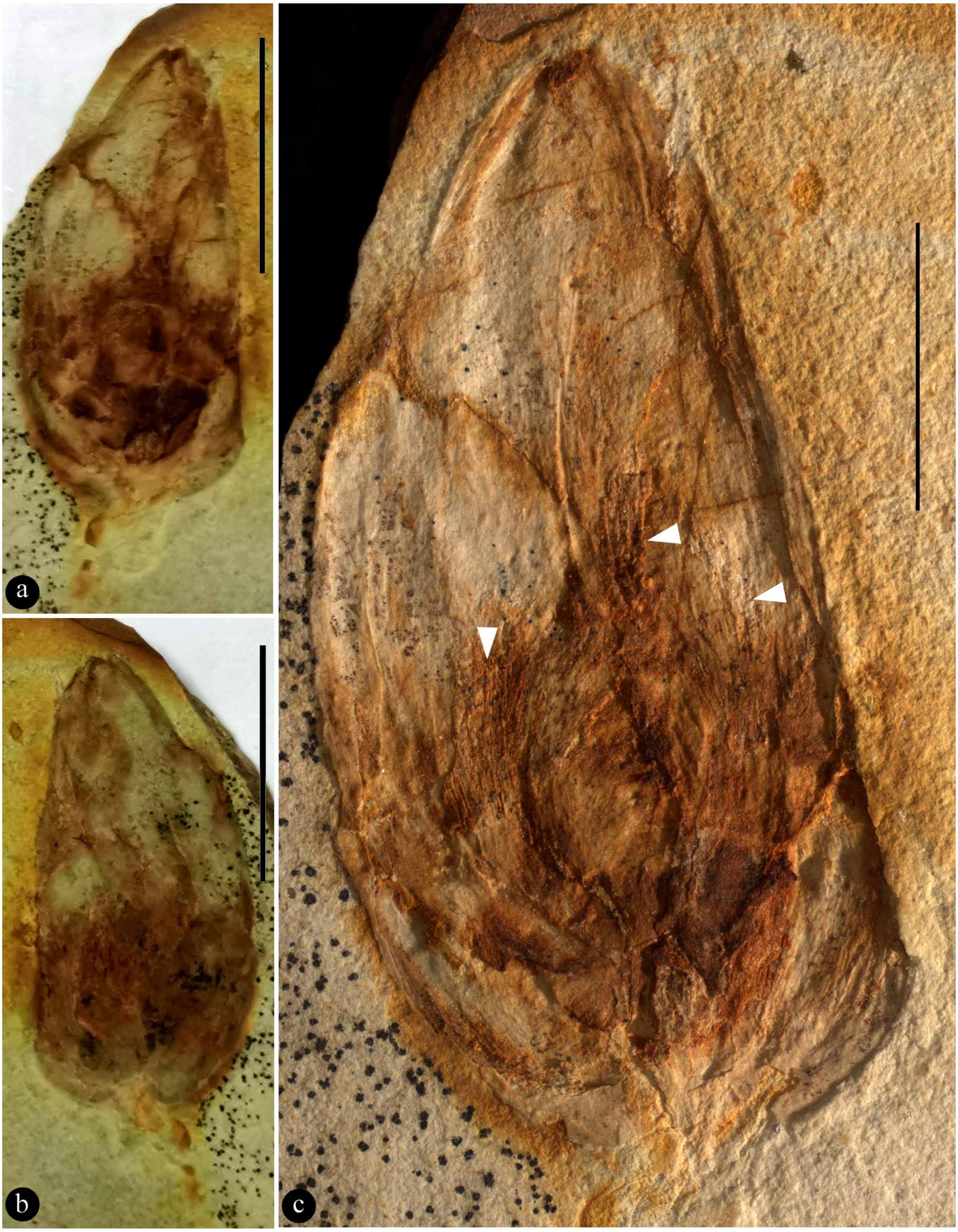

Fig. S1. More information of *Archaebuda cretaceae* sp. nov. **a-b**. Two facing parts of the same specimen. Scale bar = 10 mm. **c**. Enlargement showing details of the flower bud. Note variable textures distinguish one petal from another. Possible androecium is marked with white triangles. Scale bar = 5 mm.
